# Drug dealing and drug use prevention – a qualitative interview study of authorities’ perspectives on two open drug scenes in Stockholm

**DOI:** 10.1186/s13011-021-00375-w

**Published:** 2021-04-26

**Authors:** Kristin Feltmann, Johanna Gripenberg, Anna K. Strandberg, Tobias H. Elgán, Pia Kvillemo

**Affiliations:** STAD, Centre for Psychiatry Research, Department of Clinical Neuroscience, Karolinska Institutet, & Stockholm Health Care Services, Region Stockholm, Norra Stationsgatan 69, SE-113 64 Stockholm, Sweden

**Keywords:** Illicit drugs, Police, Situational prevention, Social prevention, Formal control, Informal control, Qualitative content analysis, Open drug scenes, Facilitator, Barrier

## Abstract

**Background:**

The use of illicit substances causes various health and social problems globally. Moreover, open drug use and dealing in urban areas, i.e., open drug scenes, can cause public order problems, lead to the recruitment of marginalized and young people for drug use or sale, and induce feelings of insecurity in the general public. Although some international studies have described various ways to manage open drug scenes, such as law enforcement and social interventions, there is limited knowledge about the facilitators and barriers promoting or impeding the implementation of such interventions. The aim of the current study was to explore how different authorities describe the nature of two open drug scenes in Stockholm and to derive authorities’ perspective on facilitators and barriers to implementing interventions to reduce open drug dealing, drug use, and related problems at these locations.

**Methods:**

Semi-structured interviews with police officers, security staff, social workers, and municipality officials (*n* = 21) in the municipality of Stockholm were conducted and analyzed by qualitative content analysis.

**Results:**

The analysis of the interviews generated the following categories: Problems, Interventions, Organizational factors, and External factors, revealing information about the strategic and daily counteracting work occurring at the open drug scenes as well as authorities’ perceptions of facilitators and barriers to implementing interventions to reduce open drug dealing, drug use, and related problems. Facilitators included motivated and skilled professionals and organized collaboration between key actors. Prominent barriers were a lack of resources to maintain personnel continuity at the scenes, policies that impede information sharing and put security staff in danger, and people who use or sell drugs without having residence permits.

**Conclusions:**

To increase the possibility of successful implementation of interventions to counteract open drug dealing, politicians and authorities should pay attention to collaboration between key actors, sufficient resource allocation, possible modification of policy governing professional duties, and remedies to the vulnerability of individuals without residence permits.

## Background

The use of illicit drugs is a recognized contributor to the global burden of disease, causing individual suffering and incurring societal costs [1]. Illicit drugs are available through various sources, such as the Internet, social media platforms, personal contacts, and public places in urban areas, so-called open drug scenes, where drugs are used and transferred more or less openly between people who sell and people who use the drugs [2].

Open drug scenes might provide an opportunity to buy and sell relatively anonymously, as the extent of sales cannot be traced over time in the same way that online transactions can. Hence, these drug scenes potentially provide a range of people, including minors and those from socially marginalized groups, with different drugs [3]. Furthermore, open drug scenes can be meeting points for socially marginalized people who might initiate or escalate drug use and dependence, leading to, e.g., infectious diseases and overdoses [4, 5]. Additionally, open drug scenes cause nuisance to the public through harassment, violence, acquisitive crime, needles used for drug injection, and prostitution, rendering them not only a public health concern but also public disturbances, creating feelings of insecurity among the general public [5, 6]. Therefore, during the past 50 years, various strategies have been developed and used in Europe and other Western countries to counteract the existence and spread of open drug scenes [5, 7–11].

Actions taken to counteract open drug scenes have ranged from ‘preventive strategies’ involving law enforcement to ‘corrective strategies’ involving social and health interventions, as described by Bless and colleagues [5]. Similarly, subsequent publications describe the use of ‘repressive’ (law enforcement) and harm reduction methods [11]. Enforcement measures refer to police interventions that can include clearance of people who use drugs from the scenes, fines for them, and imprisonment of people who sell drugs [8, 10, 11]. Harm reduction measures encompass a number of different low-threshold services that can involve providing shelter, food, clothing, counseling, needle exchange services, opioid substitution treatment, and drug consumption facilities [8]. Over the years, European cities have switched back and forth between enforcement and harm reduction measures (e.g., Oslo) [10], have moved to a more harm-reduction-oriented approach (e.g., Copenhagen) [9], or have used a combination of both approaches (e.g., Zurich) [11]. In contrast to strategies aiming to promote the coexistence of people who use drugs and the rest of society [11, 12], the counteracting strategies assessed in the current study aim to prevent, thereby reduce, and in the long run, abolish, open drug scenes. This can be seen as a natural choice based on the Swedish penal law on narcotics [13], under which drug dealing and the use of illicit drugs are criminalized. This circumstance makes public drug use a legal issue as well as a health and nuisance problem. The current strategy is based on the assumption that a reduced number of open drug scenes reduces the visible availability of illicit substances, which may reduce the risk for vulnerable groups passing by open drug scenes to be drawn into drug dealing and drug use with negative health consequences [14]. On the other hand, several researchers argue that the effect of repressive measures on the availability of drugs, such as heroin, is small [15, 16] and that these measures lead to unsafe injection practices, increasing the spread of blood-borne viruses [17–20]. Furthermore, repressive measures are thought to cause geographical or temporal displacement of drug dealing and related problems, and it is unclear to what extent these measures can actually reduce drug dealing [20]. In contrast, harm reduction methods alone do not seem to reduce the magnitude of the problem [11]. In fact, due to the complexity of the problem, a combination of prevention, enforcement, harm reduction, and treatment measures implemented via structured cooperation among different stakeholders has been identified as the most effective approach, the case of Zurich being one example [8, 11, 21]. Some researchers have even suggested coexistence between people who use drugs and the rest of society as a plausible strategy [11, 12], if accepted by the public [12]. Again, the Swedish strategy to combat illicit drugs focuses to a large extent on the protection of young or marginalized people, which may lead to other priorities than those made in countries with coexisting approaches [22].

Although some studies have described interventions used at open drug scenes either to reduce public nuisance or to prevent harm to people using drugs [5, 8–11, 21], there is limited knowledge about the effectiveness of these interventions, as well as facilitators and barriers promoting or impeding the implementation of such interventions. To assess the effectiveness of interventions, controlled trials would serve as a plausible study design. However, since this design is hard to apply on complex societal problems, researchers are often left to use e.g., qualitative methods or mixed methods designs. An important aspect to address, whether or not the effectiveness of an intervention is known, is the implementation process. A favorable implementation is crucial for a desired result and in the field of implementation research, some general knowledge on effective implementation strategies has emerged. Fixsen and colleagues compiled a comprehensive synthesis of the literature, highlighting a number of components that influence the implementation of a certain intervention: 1) the implementation process, e.g., the education and coaching of staff, 2) the organization in which the intervention is implemented, e.g., prioritization and attitudes among staff, and 3) external circumstances, e.g., societal norms, politics, and the economy [23]. Similarly, Scaccia and colleagues emphasize the importance of innovation-specific capacities, motivation to implement an innovation in an organization, and general capacities of the organization in which it is implemented [24]. To expand the knowledge on how to facilitate the implementation of interventions to counteract open drug scenes, the current study aims to explore how different authorities describe the nature of two open drug scenes in Stockholm and to derive authorities’ perspective on facilitators and barriers to implementing **i**nterventions to reduce open drug dealing, drug use, and related problems at these locations.

## Methods

### Setting

In 2017, the police authority in Stockholm County intensified the focus on counteracting open drug scenes by conducting a survey in all local police districts. The survey revealed 48 open drug scenes, defined as “geographical, permanent locations where drug use and dealing occurs openly and is perceived as problematic by authorities or the general public” [25]. Subsequently, the police authority in collaboration with the county council, the prison and probation service, the county administrative board, and the customs administration, compiled various interventions to reduce drug availability and drug demand in a ‘Methods Handbook’ [25]. As a result of the intensified focus on counteracting open drug dealing, the police districts, in cooperation with the municipalities’ social services and other key actors, such as housing and public transport companies, were advised to use the interventions to counteract the open drug scenes [7, 8, 11, 21, 26]. Even if single interventions in the handbook are not evaluated with regard to their effectiveness, they have been used internationally and can be categorized as formal control, informal control, situational crime prevention, and social prevention measures, as previously described in the literature [2, 6, 25, 27]. Formal control includes police interventions, such as foot patrolling, control of vehicles, and camera surveillance. Formal control is thought to assist with not only the detection and prosecution of crimes but also their prevention. Informal control is thought to be achieved through the increased presence of citizens who do not engage in drug use or dealing. Situational prevention is aimed at reducing the use of the location for open drug use and dealing by making changes to the environment, e.g., the physical characteristics of a square, which should be initiated through collaboration among different local stakeholders. Social prevention is aimed at reducing the motivation to commit crime, here referring to drug use, dealing, and related crimes. These interventions usually involve social services and include, for example, an obligatory, formal report of concern regarding a minor whose welfare is at risk (e.g., due to drug use). Another example is an intervention where a police officer drives a minor who is suspected of drug use to a health care facility where the minor can be tested for drug use and obtain further help regarding addiction treatment through the involvement of parents, healthcare providers, and social services (i.e., the so-called MUMIN method (Maria Ungdom Motiverande Intervention (In Swedish) Maria Youth Motivation Intervention) [25]. The methods in the Handbook were used also before 2018, but the compilation of them in a handbook along with intensified efforts and collaboration to combat open drug scenes at the selected locations, was expected to reduce open drug dealing and drug use in these areas.

### Choice of locations

The research team planned the current study in collaboration with the local police authority in Stockholm, which provided information on the characteristics of the open drug scenes in the county and how the counteracting work was carried out at these locations. Subsequently, the research team selected two locations for analysis: one at the city center (central scene) and one in the outskirts of the city (suburban scene), both being squares adjacent to a subway station. The work targeting both scenes was, according to the police authority, prioritized by the local police districts and Stockholm municipality, and a clear structure of collaboration between these actors and several other local stakeholders was already established, which was one of the criteria for the selection of the scenes. However, whereas the central scene has been known nationwide as an open drug scene since the 1960s and is surrounded by many cafés, shops, and businesses, the suburban scene is located in a residential area of low socioeconomic status and was detected as an open drug scene only a few years ago. Furthermore, the police have been actively implementing methods presented in the handbook at the central scene since 2018, whereas the suburban scene started to be prioritized by the police in 2019. An ambition to include different types of locations to get a rich material regarding facilitators and barriers for implementation of counteracting methods, further guided the research team when selecting these two scenes.

### Interviews

The research team conducted semi-structured telephone interviews with key informants involved in the work at the scenes. The choice of telephone interviews instead of face-to-face interviews was based on an ambition to make the interviews time efficient for the informants. To recruit informants, the team employed purposive sampling to select professionals working in connection with the scenes, with the aim of including 20–30 informants to achieve reasonable saturation [28]. In early summer 2019, the research team contacted professionals in organizations involved in the work at the selected drug scenes via e-mail, asking them to participate in the study. Leading officials in the organizations provided the researchers with contact information to individuals/professionals within their organizations who had great experience of the counteracting work at the scenes. Since the research team did not have direct contact with all potential informants, there are no information on refusal, e.g., number of people asked who did not want to participate. The researchers provided potential informants with information on the aim of the study, i.e., to evaluate the counteracting work at the open drug scenes, that participation was voluntary, that the telephone interviews were to be recorded with permission, and that the interview material was to be compiled so that no single informant could be identified. The research team obtained informed consent by asking the receiver to reply to the e-mail, agreeing to participate in the study. If recipients refused to participate, the researchers contacted additional persons from the same vocational group. The final group of informants (*n* = 21) included police officers (*n* = 11), security staff (n = 2), outreach social workers (*n* = 4), and officials from the municipality (n = 4). With the exception of the security staff, representatives from both the ground and management levels were selected for interviews. None of the municipality officials were politicians or appointed by politicians. The informant group consisted of 11 men and 10 women. After the vocational categories were determined, the research team elaborated semi-structured interview guides for each vocational category. The interviews included questions about drug dealing and related problems at the scenes, interventions used to counteract these problems, facilitators and barriers influencing the implementation of the interventions and the informants’ views on what is required to abolish open drug dealing at the scenes. Two of the authors (KF, PK) conducted the interviews, which were on average 41 min long (range 20–120 min). After 21 interviews had been conducted, KF and PK perceived that no or little new information could be obtained by additional interviews, and subsequently terminated the interview process. Recorded audio files were saved under a code name, and the interviews were transcribed verbatim.

### Qualitative content analysis

Qualitative content analysis is a flexible family of methods for the analysis of qualitative data, covering impressionistic, intuitive, interpretive, as well as strict textual analytical approaches [29]. Content analysis provides researchers with a usable tool that can be used in a more inductive form (conventional), if theories on the research topic are lacking, as well as deductive form (directed) when theories to build on are present. Since previous research has detected a number of factors (facilitators and barriers) which tend to appear when various organizations introduce new routines or methods [23, 24], the research team found that a more deductive form of content analysis (directed) was a plausible choice of analytical method. Additionally, the researchers employed a team-based approach [29–31], and utilized NVivo 12 software to structure the data to ensure the credibility of the results [32]. The initial reading of the material was to a large extent guided by concepts outlined in the background section, i.e., training and coaching of staff, prioritization, attitudes and norms among staff, societal norms, politics, and economy [23], innovation-specific capacities, motivation, and general capacities of the organization in which the method or routine is implemented [24]. Initially, two researchers (KF, PK) started the analysis process by repeatedly reading interviews from various vocational categories, trying to get an overview of the material. During the reading of initially eight interviews, which the researchers required to get acquainted with the material, KF and PK identified meaningful units and grouped them into preliminary categories and codes, as exemplified in Table 1.

**Table 1:** Example of analysis

Meaning unit	Condensed meaning unit	Code	Category
*Youth at risk are always prioritized because there is a chance to prevent future addiction.*	The motivation to support young people at risk is high.	Motivation	Organizational factors

Based on the initial reading, KF and PK developed a preliminary coding scheme which contained four categories (problems, interventions, organizational factors, and external factors) along with several codes with definitions (codebook) [31] and presented it to the other researchers in the team who approved it. Using the preliminary coding scheme, KF and PK read the rest of the interviews and found that the coding scheme overall captured the content in the interviews, however not perfectly. When discussing their new findings, KF and PK resolved some issues by commonly focus on potential new definitions of some of the codes, resulting in the elimination of one of the codes and the division of another into two. The research team subsequently agreed to adopt the final coding scheme, as outlined in Table 2, and KF and PK completed the coding of all interviews again with satisfactory result, i.e., no new revision of the scheme was needed.

**Table 2:** Coding scheme

*Categories*	Problems	Interventions	Organizational factors	External factors
*Codes*	Drug use	Formal control	Motivation	Place characteristics
	Drug dealing	Informal control	Resources	Socioeconomics
	Target group	Situational prevention	Collaboration	Immigration
	Criminal activities and disturbances	Social prevention	Culture	Civil society
			Working environment	School
			Knowledge	Laws and regulations

To further ensure the trustworthiness and credibility of the results, the research team provided a Swedish compilation of the study findings to the informants.

## Results

In the following section, the results from the content analysis are presented in accordance with the four identified categories.

### Problems

#### Drug use

According to the informants, drug use occurs at both the suburban and the central scene, although an increased presence of police officers seems to have recently diminished public drug use and overdoses at the central scene. Drug use, especially injecting, does not seem to occur visibly at the central scene but rather in its proximity or in enclosed rooms (e.g., restrooms). According to the informants, older users have moved from the central scene to two other areas of the Stockholm city where needle exchange services are located. One of the informants meant that open drug use (apart from the smoking of cannabis) at the suburban scene is less common due to the substances dealed at the place, i.e., primarily cannabis, tramadol and cocaine, the two latter not immediately used by the buyer.

#### Drug dealing

Both drug scenes are publicly known places for illicit drug transactions. At the central scene, cannabis products, prescription opioids, benzodiazepines and heroin can easily be obtained by anyone, according to the informants.

*Anyone can at any time basically go down to [the central square] and get drugs.*

(Police officer 3)

The presence of uniformed staff has, according to the informants, reduced open drug transactions, especially at the central scene, but contacts between people who sell drugs and people who buy them continue, with actual transfers carried out elsewhere. According to the informants, drug dealing at the suburban scene, indoors and outdoors, still occur quite openly, although the presence of uniformed staff can temporarily prevent visible transfers. One of the informants described the situation as follows:

*There is a restaurant that for many years has been a place where criminals hang. They [people who sell drugs] use it as their own living room. When the police are not there, they sit there and do both drug dealing and socialize. And when the police come, they leave, and then they’re back when the police are not there. So they have somewhat taken over the place, so to speak. And then if you look out in the square, and connected to the subway, there is dealing going on.*

(Police officer 5)

#### Target group

According to the informants, several of those who visit the central scene are people troubled by psychiatric or socioeconomic problems and are sometimes drawn to the square out of loneliness. Additionally, young people from neighboring communities and from other parts of Sweden use the central scene as a meeting point. People who sell or use drugs at the drug scenes are primarily teenagers and young adults, according to the informants. Older people who use drugs, previously present at Sergels torg, have moved away to other parts of the city and the scene is, according to the informants, dominated by male dealers from three ethnic groups. However, young hang arounds, both girls and boys, who are attracted to an “exciting” place are also present at the scene. According to the informants, the suburban scene, in contrast to the central scene, is dominated by one criminal gang.

#### Criminal activities and disturbances

In addition to drug dealing, both scenes are according to the informants characterized by other criminal activities, such as violence or threats of violence, robbery, and theft, mainly pickpocketing and shoplifting. Shop and restaurant owners, as well as security staff at the suburban scene, are threatened and the dominance of one gang at this location leads to higher rates of threats and fear and subsequently lower levels of reported violence, according to the informants. Young people at the suburban scene shoplift openly, hide stashes of drugs in stores and in general ‘act as if they own the place’, according to the informants, and it is mostly local boys who handle the drugs, supervised by older individuals, since children under the age of 15 years cannot be held accountable by law.

*Young boys, maybe 12–14-year-olds, are those who handle the drugs, deliver and carry the drugs, because they [young boys] know they [young boys] have not reached the age of criminal responsibility.*

(Police officer 3)

As mentioned above, three criminal networks compete for the drug market at the central scene and violence occurs both between the criminal groups and between those who sell and those who buy drugs, e.g., the incorrect substance or amount of money is handed over, according to the informants. However, the relatively strong presence of police officers and security staff prevents the escalation of violence and prevents either of the criminal networks from achieving dominance over the area, according to the informants.

### Interventions

#### Formal control

Uniformed police officers carry out formal control, maintaining order by intervening when arguments or violence arise between criminal groups and by preventing drug dealing and other crimes, as described by the informants. On suspicion, the police search people for drugs and report possession or dealing. Moreover, they search for drugs at typical hiding places around the square, sometimes using trained dogs and also remove violent or visibly intoxicated people from the squares, according to the security guards and the police officers interviewed. Police officers in plain clothes are, according to police officers, stationed at the squares to observe and intervene when they witness individuals dealing drugs. At the central scene, they also use surveillance cameras while collaborating with officers in uniform. Both uniformed and plain clothes police officers aim to visit the scenes between other tasks and engagements, which, according to themselves, leads to varying levels of police presence at the scenes. While there is a daily presence of police officers in uniform at the central scene, this presence is much lower at the suburban scene, according to police officers and security guards. At both locations, reconnaissance is important for obtaining knowledge about strategic individuals in the criminal networks, as stated by police officers who also described house searches where the police can seize drugs, leading to the prosecution of those individuals. Profiling individuals by observing them is also used to gather evidence and create a legal basis for house searches, according to the police officers. Formal control at the scenes is also carried out by security staff. The role of the security staff is to maintain order and prevent crime, including drug dealing, as described by one of them as follows:

*Our task is to disturb the dealing. We must be visible, and through our visibility we disturb the [criminal] networks.*

(Security guard 1)

In contrast to the police officers, the security staff are not allowed to search people based on suspicion of possessing or dealing drugs, but they can search for weapons, according to themselves. Other forms of supervision, mentioned by the informants, are undertaken by adults, such as social workers, workers from volunteer organizations, and youth club personnel.

#### Informal control

According to the informants, the central scene has many passers-by, primarily during the daytime, however, people rarely stop at the scene during their walk to their destinations. During summer and vacation, more youths, marginalized people and people dealing drugs are present in the square compared to the winter season. At the same time fewer police and security staff are working, as described by the informants. To increase informal control and compensate for the shortage of formal control during summertime, a program with many cultural activities was conducted during the summer of 2018 at both scenes. According to one of the informants, this contributed to reduced drug dealing and related problems.

*We have not had such a calm period in years, and when I take a look at the crime statistics, I observe a decrease during the summer months, compared to previous years.*

(Municipality official 5)

#### Situational prevention

Representatives from the police, the municipality, the property owners and other organizations meet regularly to discuss situational prevention at both scenes, according to the informants. At the central scene, better lighting and the removal of possible hiding places for drugs, such as bags around tree trunks or window ledges, have been discussed, and recurrent actions were described by one informant as follows:

*… fixing doors that are not working, installing locks where they were missing. One keeps the area clean and tidy continuously. Just now, the cleaners who go around every day, assigned by the [municipality] business association, give a whole different effect. [The square and surroundings] are today tip-top.*

(Police officer 7)

As described by one of the informants, the square at the suburban scene was planned to be rebuilt during 2019/2020, with the police having an advisory role in the process. For instance, the police advised that the square be surrounded with high trees instead of low bushes, with the aim of obstructing drug dealing and other criminality by improving the surveillance possibilities.

#### Social prevention

To prevent the development of substance use disorder among youths and hinder them from becoming involved in drug dealing or other criminal activities, police officers and social workers approach adolescents at the scenes and attempt to persuade them not to spend time at the squares and to accept help if needed*.* Upon suspicion of drug use among minors, parents are contacted according to the informants. Social workers also described that they visit schools to present themselves and offer support to students. If concerned for a minor’s welfare, police officers and social workers file the legally required formal reports with social services and one of the social workers of the youth outreach service reported having meetings involving the minor, the parents, and social services. The MUMIN method, described in the methods section, was mentioned in a positive way as an opportunity for the minor to receive immediate attention from healthcare professionals.

*And MUMIN is when one interrogates an adolescent being suspected of maybe a minor drug offense, own use or something, that we are there and have a social talk and make a report to the social services. Often, when you go to [the youth addiction clinic], you can directly meet a doctor and get a doctor's assessment and an appointment for a follow-up.*

(Social worker 4)

### Organizational factors

#### Motivation

The detection of open drug dealing seems to be viewed as important by all the professionals, and most of the informants expressed a high level of motivation to come to terms with open drug dealing and its related problems, especially the recruitment to use or dealing.

*If we can arrest many people who sell, then the narcotics disappear from the streets and the access to it and the recruitment to the business will be reduced.*

(Police officer 4)

Informants from both scenes also mentioned efforts to increase use of the MUMIN method but emphasized that it is very time consuming and therefore difficult to apply to every relevant case. At the central scene, the use of MUMIN is quantified, and clear objectives for its use are set, according to one of the social workers. Also, informants connected to the suburban scene expressed motivation to start using MUMIN. Although cooperation between organizations and professionals involves considerable effort, the need to ‘work together’ and address the problem from ‘all angles’ was mentioned by several of the informants. However, a constant replacement of people prosecuted for dealing causes frustration among police officers and tends to reduce their motivation. Additionally, the reorganization of the Police Authority in 2015 resulted in a decrease in police officers focusing specifically on drug criminality, which, according to one of the informants, created feelings of having to ‘invent the wheel time and time again’.

#### Resources

Although the prioritization of work at open drug scenes seems quite high among the actors involved, a lack of resources in the police organization was mentioned by several informants, including police officers, security staff, and municipality officials. For example, due to activities at other locations, such as demonstrations or criminal incidents, the time and personnel the police allocate to the scenes are deemed insufficient, which also people from other vocational categories than the police regret. The lack of police officer continuity at the scenes impedes the development of knowledge about target individuals, according to the informants, and the recent dissolution of specialized task force units working against, for instance, drug-related crimes was described as follows:

*What works best of all is having police officers in the outer service who can work directly in a specific area. [ … ] But it has not been like this since the merging of the [local police] authorities in 2015. Since then, all policemen have to do everything instead of working specifically with problem areas. Many specialized task force units have been destroyed.*

(Police officer 4)

A need for more plain clothes police officers, who are less visible and thereby able to detect drug dealing at the scenes, was also brought up by the informants. In addition, more personnel resources are perceived as needed to enable more effective use of surveillance cameras.

*Yes, we use camera surveillance [ … ] but we have not yet found a working structure for this and have not allocated sufficient resources.*

(Police officer 1)

#### Collaboration

Regarding collaboration, police officers, security staff, and outreach social workers report an effectively functioning cooperation between their organizations and staff, also formalized in written agreements. Especially at the central scene, regular meetings are held in the common meeting room located at the square, wherein information about individuals and problems is shared and activities are planned accordingly. The sharing of information facilitates the work and prepares different professionals for situations that can occur at the drug scenes, as expressed by one of the social workers as follows:

*Often, we get information about the seizure of illicit drugs, which has led to a reduction in, for example, heroin at the scene, which can create unrest and a need for caution. Such information is very important for those who do outreach work.*

(Social worker 4)

Collaboration in the form of day-to-day work as well as more strategic planning was mentioned as important. Attempts to involve healthcare actors in the collaboration forums have been unsuccessful thus far. Some of the informants also expressed a desire for more collaboration with schools, parents, and businesses.

#### Culture

The cooperation between the different organizations seemed to be characterized by a culture of generosity and supportive relations. A common room for planning and having coffee at the central scene has contributed to a good collaborative climate, according to several of the informants.

*To get to know each other is important for a good collaboration climate, and this room has really contributed to that.*

(Social worker 4)

Despite this, daily cooperation is sometimes perceived as dependent on certain individuals, as described by one of the security guards as follows:

*The cooperation with the police varies to a great extent depending on where you are and, unfortunately, is down to an individual level.*

(Security guard 2)

Given that cooperation depends on certain individuals and is therefore unstable, informants stressed the importance of priorities and mandates from higher up in the respective organizations as well as clear structures for responsibility, indicating that these are still lacking.

#### Working environment

The working environment at the squares seems to vary between professions. Security staff expressed that they were in a vulnerable position when approaching criminals at the scenes due to a lack of education, restricted access to information about individuals due to secrecy regulations, a lack of rooms in which to keep persons whom they take into custody, and a lack of anonymity when having to testify against criminals in court. One of the security guards described the high degree of exposure accompanying court appearances as follows:

*Every time you testify or are a plaintiff, you appear with name, social security number, address and everything. This creates an exposed situation, and it feels like the legislation is lagging behind.*

(Security guard 2)

Other professionals did not note working environment problems to a large extent but praised the collaboration between professionals, which they thought contributes to a more secure working environment.

#### Knowledge

Knowledge about the methods in the handbook seems high among the informants and several police officers who, according to themselves, have worked to counter drug crime for many years mentioned various actions that can be taken to combat the drug scenes, including those stated in the handbook. Moreover, mobilization and systematic work based on strategies and goals appear to have contributed to a joint understanding of the situation among the key actors involved, as expressed by a police officer who had worked in the area for 15 years.

*What distinguishes 2019 from 2006 is that we now have a much deeper, broader, more adequate picture of the situation. We have a much greater understanding that we cannot work with just one thing at a time, but we must work broadly.*

(Police officer 2)

The sharing of information among the professionals regarding the ever-changing situations at the drug scenes but also regarding the various professions’ roles, responsibilities, and work conditions, was emphasized as important in building a common knowledge base. However, high staff turnover in certain organizations challenges the ability to maintain knowledge and competence, especially among security staff, according to one of the social workers. One of the police officers also underlined the importance of specialization to achieve sufficient knowledge to carry out the tasks effectively.

*If you work with narcotics, you get skills concerning both substances, what to look for, etcetera [ … ] because, there are trends in this business. So, if you want to be really skilled, you may have to work solely on this problem during a period, instead of changing between different tasks all the time.*

(Police officer 5)

### External factors

#### Place characteristics

Very few people live in the area where the central scene is located, as described by the informants who meant that people are passing by primarily in day time. Conversely, many people live in the suburban scene, but without engaging very much in events related to the open drug scene, according to the informants. During the daytime, the central square is an important place for recurrent demonstrations and speeches. However, the square is also perceived as a meeting place for people who are excluded from mainstream society according to one of the informants, who stated that loneliness is one of the reasons they visit the place.

*There are so many lonely people. I actually want to point out that I have talked to many people there [at the square] who just stand there trying to get in contact with someone who wants to talk to them.*

(Police officer 10)

One of the informants described a 24-h fast food restaurant as meeting plac for people who create messes and start quarrels, creating an unsafe environment for people passing by. At the suburban drug scene, the physical layout of the square contributes to a general impression of insecurity, and problems with pigeon droppings and graffiti, according to the informants, adding that the square also has many hiding places, which facilitates drug dealing, and the need for a brighter, more open space was expressed. Furthermore, groups of people with alcohol use disorder was described as visiting the square due to the adjacent alcohol retail outlet and another organization providing activities for this target group.

#### Socioeconomic status

Several informants highlighted socioeconomic factors as contributing to the recruitment of people to drug dealing, thereby influencing the size of the problem to be handled. The low socioeconomic status characterizing the suburban scene entails high rates of unemployment, which some informants said made it a hotbed for the recruitment of young people to criminal networks. To prevent this, police officers and social workers suggested that education and job opportunities, as well as organized leisure time activities, are needed. One of the informants expressed the following:

*The society should get people employed. Instead of hanging around, they should do meaningful things during the day.*

(Police officer 4)

Although there are some clubs offering leisure activities, high participation costs and the difficulties entailed in starting at a later age were mentioned as barriers for adolescents. One informant expressed the wish that more local businesses would offer jobs to young people.

#### Immigration

According to the informants, many immigrants, some without residence permit, are present at the central scene, making them especially vulnerable to recruitment into drug dealing.

*Those whose asylum applications have been rejected. [ … ] They have nothing to lose, and they know that they are not welcome in their country either maybe. And they are not allowed to be in Sweden. They are not allowed to work.*

(Police officer 10)

Although people at risk of evading deportation can be arrested, one of the informants said that few secured housing places are available for this purpose. Furthermore, the informant pointed to the fact that minors who are not granted asylum can go to high school but are not allowed to work, which the informant meant could put them in a vulnerable situation as it is hard for them to support themselves legally. Immigration has increased in recent years and has posed new challenges to the police, as expressed by one of the police officers who said that there is a need for better cooperation with the migration board and the border police to discourage criminal activity. Regarding social prevention methods, there is a lack of support from social services for drug-using foreign minors and a lack of engagement by the legal guardians of unaccompanied minors, according to a police officers, who also meant that many of these adolescents have been assigned to housing in northern Sweden but prefer to stay with friends in Stockholm, putting themselves in vulnerable situations and making it difficult for the authorities to help them.

#### Civil society

Several of the informants reported that they have noticed the emergence of a more liberal view on drugs and a normalization of drug use in society, especially with respect to cannabis, which they thought contribute to an increase in drug use. Moreover, fear of revenge from criminals prevents locals who live near the suburban drug scene from intervening in drug use and dealing, according to one of the informants, stressing the importance of engagement by the civil society.

*We need to engage the local residents [...] to be part of something bigger, and that platform we have to create now*.

(Municipality official 5)

Reaching out to parents is considered central in activating the local community, according to several of the informants who consider patrolling at, and around, the open drug scenes by parents and other adults as an important means to render the area more secure. However, parents’ attendance at patrolling evenings organized by the school has so far been low, according to one of the municipality officials.

#### School

Schools are identified by some informants as an important potential actor in the work against open drug scenes. Several schools around the suburban scene report adolescents dealing drugs on school grounds, according to one of the informants connected to the suburban scene. However, after a dialogue between the schools and the police, a common letter was sent out by the heads of twelve schools informing parents about the problem and calling for patrolling. A police officer at the central scene mentioned similar attempts to initiate such actions there. However, another police officer expressed that the schools do not take seriously their legal responsibility to report concerns about students’ welfare regarding, for example, drug use to social services. Social workers, however, try to raise the problem and suggested possible actions that can be taken during meetings with the schools.

*We try to make contact with schools and meet with them and talk [ … ] about how we can complement each other and work supportively for the kids.*

(Social worker 1)

#### Laws and regulations

Several informants brought up issues related to laws and regulations, which they felt sometimes impede the effective handling of problems at the drug scenes. Some of them claimed that the law mandating professional secrecy, especially in social services, impedes collaboration by the different professions when attempting to support adolescents. One informant, however, said that sometimes closer collaboration and information exchange was possible between the social services and the police after a mutual agreement to be less strict concerning professional secrecy was made. Other regulations impeding the work at the scenes are policies governing the tasks of security staff, e.g., restrictions on searching people for drugs based on suspicion, limited access to information about criminals, and the lack of anonymity when witnessing in court, as mentioned above by one of the security guards. This is especially troublesome since some exclusive warrants of the police, e.g., the monitoring of demonstrations, often lead to security staff taking over the role of the police, as expressed by one of the police officers.

*We want to work in these environments [..] but we stand mostly at some demonstrations today. All kinds of weird gatherings that we have to follow through the city for several hours. And then, the security staff drive around as ‘police officers’ and we stand at some traffic crossing and direct the traffic.*

(Police officer 10)

Minors can be arrested for drug use or dealing but are usually not detained by the prosecutor, according to one of the informants who also meant that this practice affects migrants without IDs who claim to be underage and are arrested for drug dealing. The police expressed a need to speed up the prosecution process regarding drug crimes through mandatory collaboration between authorities to effectively counteract drug dealing. Additional policies affecting the work of the police at the drug scenes are that girls can be searched only by female police officers (of which there are few), that they cannot expel people who are known as sellers of drugs from the scene if they are not disturbing the public order or have no drugs on them, and that the regulations do not allow having a person to watch the surveillance cameras around the clock, according to police officers. One of the municipality officials informant also mentioned that social interventions cannot always be initiated if the person of interest is present regularly at the square but has his/her residency in another municipality.

## Discussion

The present study examined one suburban and one central open drug scene in Stockholm, Sweden, focusing on authorities’ description of the nature of the scenes as well as their perspective on facilitators and barriers to implementing interventions to reduce open drug dealing, drug use, and related problems at these locations.

### The nature of the scenes

Both the central and the suburban scenes seem to fit the descriptions from earlier studies on open drug scenes, with drug use, dealing and other criminal activitiecs and nuisance taking place [5, 8]. However, in contrast to drug scenes in cities in neighboring countries, such as Oslo [10] and Copenhagen [9], and even past European drug scenes [11], the Stockholm central scene does not seem to attract many people who use heroin. Some heroin-dependent individuals who used to visit the scene have now moved to the two areas in Stockholm where needle exchange services are offered. Whether this circumstance influences the implementation of interventions targeting other risk groups is not clear from this study, but the low rate of visible injection use may decrease feelings of insecurity at the site and thereby perhaps increase public visits and strengthen informal control. As this study do not investigate the effects of interventions on people who use drugs, no information about current informants’ or health care staff’s perspective on this issue was collected. From a public health perspective, the investigation of health-related consequences among people using drugs and their relatives is of course important, as is also the underlying ambition of the combat of open drug scenes, i.e., to prevent young or marginalized people from being drawn into drug use and dealing (with subsequent health related problems), and should preferably be included in future studies. Drug dealing at the two scenes is conducted by well-organized criminal networks using people below the age of criminal responsibility at street level, which highlights the importance of protecting minors from visiting the current scenes and of detecting senior people who sell drugs with methods other than direct observation of dealing. Formal control in the form of intensive patrolling seems to have changed the central scene from open to more closed, i.e., with drug transactions taking place elsewhere [26]. This circumstance may contribute to a reduction of visible signs of drug dealing and perhaps a safer impression of the place while also implying that people who sell drugs can escape detection when handing over illicit drugs.

### Facilitators of the implementation of interventions

The elaborated structure of collaboration between the police and other organizations and stakeholders, formalized in contracts between the heads of the organizations [25], appears to be a prominent facilitator of the implementation of counteracting interventions, as confirmed in previous studies in various European cities, such as London, Dublin, and Zürich [7, 11, 26]. The formalization in written agreements, along with the regular meetings of the professionals from the organizations involved, indicates that the work to counteract open drug scenes is prioritized among key actors in accordance with what is required for successful implementation [23]. However, several of the informants believe that additional resources are required to ensure personnel continuity among police officers and security staff at the current locations, suggesting that the counteracting activities at open drug scenes actually need to be given even higher priority. Motivated and skilled professionals appear to constitute an additional facilitator for the implementation of interventions, in line with previous research [23, 24]. Additionally, a culture characterized by generosity and understanding between organizations seems to facilitate this common effort. Most of the informants expressed enthusiasm for their work and concern over the situation at the open drug scenes, not least for those at risk of being drawn into using or dealing. Professional knowledge about the problem and a shared view of what needs to be done also appears to facilitate the implementation of counteracting interventions. However, lack of personal continuity at the scenes and with regard to performance of specialized tasks imped the ability to achieve high levels of knowledge and skills to a certain extent. Finally, the organized collaboration among the police, housing companies, and public transport companies appears to facilitate situational prevention activities, such as regular cleaning and permanent physical changes of the squares. These activities can increase informal control through efforts to make the two locations attractive, in accordance with the ‘broken windows’ theory, which states that visible signs of crime, anti-social behavior, and civil disorder create an urban environment that encourages further crime and disorder [27, 33].

### Barriers to implementing interventions

A barrier to effective formal control at the open drug scenes is the lack of resources to maintain the continuity of uniformed and plain clothes police officers. Competing police tasks often leave security staff alone at the scenes with a limited mandate to intervene against drug dealing and other criminal activities. Additionally, the use of cameras for surveillance, previously highlighted as an effective tool to observe drug transactions [34], is not fully implemented due to being too dependent on personnel resources, according to the police. Another barrier affecting the cooperation between professionals, e.g., between the police and social services and between the police and security staff, is confidentiality rules that impede the exchange of information about people who sell or use drugs. The Swedish law on public access to information and secrecy [35] states that secrecy applies to authorities’ information on individual’s personal situation if avoidance of harm for the individual cannot be guaranteed. This strict approach can contribute to an unsafe work environment for the security staff, since they have less or no information about the individuals they have to approach. With regard to information exchange between the social services and the police, the law on public access to information and secrecy allows certain exceptions, e.g., if the social services have information about individuals younger than 21 years of age and perceive that the individual is at risk of committing crimes. One of the informants mentioned that in a previous position, information sharing between the police and social services had taken place to a larger extent than in the current situation, indicating that it might be possible to meet the need for information sharing between the social services and the police authority without having to extensively revise the law on secrecy. Moreover, rules governing the procedures in court do not allow anonymity for security staff, as described by one of the security guards who meant that this circumstance put security staff in an exposed position.

In parallel with the direct prevention of open drug dealing, social interventions to prevent additional recruitment to dealing and using illicit drugs, thereby protecting individuals from addiction and reducing the demand for drugs, were also described by the informants. Even if the number of people who sell or use drugs per se is not a facilitator or barrier to the implementation of counteracting interventions, it determines the size of the task and the number of resources required. With regard to social interventions, there are barriers connected to circumstances in which individuals in need of support lack residence permits in Sweden or are minors with parents abroad. Social support for minors often requires the engagement of parents, which can be problematic in the case of unaccompanied, underage immigrants. Additionally, adult immigrants without residence permits are in a vulnerable situation because they lack work and income, which can lead to their recruitment into drug dealing. There are also several external factors challenging the counteracting of open drug scenes. Few people live around the central scene; therefore, informal control by residents is lacking. In contrast, the suburban scene is located in a residential area. Thus, on the one hand, there is potential to engage the local community to make the place safer, but on the other hand, the criminal network can control shop owners and citizens, who seldom dare to report crimes. Several informants also described the lack of engagement by the school and parents in strengthening informal control and preventing the use of school grounds as hiding places for illicit drugs. In addition to factors more directly impeding the counteracting of open drug scenes, the informants described socioeconomic factors contributing to an individual’s involvement in the drug trade, pointing to social problems, limited job opportunities, and loneliness among certain groups of people. Furthermore, the lack of work for immigrants without residence permits could constitute a risk for recruitment into drug dealing activities. Finally, an increasingly liberal view of drug use in society, observed by informants in the current study and confirmed in European surveys [36], may contribute to extended demand driving drug supply and dealing activities.

### Strengths and limitations

The current study has several strengths. First, the selection of two open drug scenes located in areas with different functions and socioeconomic statuses provides a broad understanding of the factors influencing the counteracting work. Second, the inclusion of informants from four professional categories with representatives from both the street and management level revealed different experiences and conflicting interests, which deepens the understanding of the implementation process. Third, the trustworthiness and credibility of the study results was ensured by a team-based analytical approach and the provision of possibility for the informants to read a compilation of the study results, the latter resulting in no informant pointing out inconsistencies between what was stated in the interviews and the results presented. Fourth, the authors believe that the study results to a significant extent are transferable and conformable to other international contexts, since there are similar collaborative initiatives taken in, e.g., open drug scenes in European cities [7, 11, 26]. However, there are also some limitations. The qualitative design involving recruitment of informants on a voluntary basis entails a risk of selection bias, which may lead to the collection of material not representative of all the professionals involved in the counteracting work. Moreover, although the informants were anonymous in relation to their organizations, there is a risk that they have given answers believed to be socially acceptable [37]. Finally, the current study does not assess the effects of the strategy and interventions used, limiting the understanding of the potential of the interventions actually carried out. Future research could combine long-term qualitative and quantitative data on both interventions and outcomes to provide a deeper understanding of effective prevention of open drug dealing.

## Conclusions

To increase the possibility of successful implementation of interventions to counteract open drug dealing, politicians and authorities should pay attention to working collaborations between key actors, sufficient resource allocation, possible modification of policy governing professional duties, and remedy of the vulnerable situation for individuals without residence permits. To obtain a deeper understanding of effective prevention of open drug dealing, there is a need for studies combining long-term qualitative and quantitative data on both interventions and outcomes.

## Data Availability

Collected data will be available from the Centre for Psychiatry Research, a collaboration between the Karolinska Institute angd Region Stockholm, but restrictions apply to their availability, as they were used under ethical permission for the current study and so are not publicly available. However, data are available from the authors upon reasonable request and with permission from the Centre for Psychiatry Research.

## Abbreviations

MUMIN: Maria Ungdom Motiverande Intervention (In Swedish) (Maria Youth Motivation Intervention)
STAD: Stockholm prevents alcohol and drug problems
